# Genetic and phenotypic differentiation between invasive and native *Rhododendron* (Ericaceae) taxa and the role of hybridization

**DOI:** 10.1002/ece3.38

**Published:** 2011-11

**Authors:** Alexandra Erfmeier, Marina Tsaliki, Christel A Roß, Helge Bruelheide

**Affiliations:** 1 Institute of Biology/Geobotany and Botanical Garden, Martin Luther University Halle-WittenbergAm Kirchtor 1, D-06108 Halle, Germany; 2 Vegetation Ecology and Conservation Biology, Institute of Ecology and Evolutionary Biology, Bremen UniversityLeobener Street, D-28359 Bremen, Germany

**Keywords:** AFLP, frost hardiness, germination, introgression, RGR, *Rhododendron* section *Pontica*

## Abstract

Hybridization has been repeatedly put forward to explain the invasiveness of *Rhododendron ponticum* L. in the British Isles. The present study investigates the pattern of ecotypic differentiation and hybridization among native North American *R. catawbiense* and *R. maximum*, native *R. ponticum* from Georgia and Spain, and invasive *R. ponticum* from Ireland and aims to assess the contribution of hybridization for *Rhododendron* invasion in the British Isles. Six populations per taxon were analyzed with AFLP markers for genetic dissimilarity, subjected to germination and growth experiments, and tested for frost hardiness. We assessed variation in morphological and ecological characteristics to identify traits displaying evidence of hybridization, thus, promoting invasiveness. Molecular marker analyses revealed a clear distinction between North American *R. catawbiense* and *R. maximum* on the one hand, and all *R. ponticum* populations on the other hand, displaying a complete intermixture of native Spanish and invasive Irish populations. Multivariate analyses of traits revealed leaf length–width ratio, relative growth rates (RGRs) in leaf length, root biomass, and shoot–root ratio to significantly discriminate between the different taxa and unequivocally assigned invasive Irish *R. ponticum* to the Spanish phenotypes. While the Irish *R. ponticum* had similar growth traits as conspecific native *R. ponticum* provenances, germination and biomass allocation were more similar to North American *R. catawbiense* and *R. maximum*. Hybridization did not contribute to explaining invasiveness of *R. ponticum* in Ireland. The similarity in germination and biomass allocation of invasive Irish *R. ponticum* and North American species has evolved independently and can more probably be attributed to an independent shift within the Ponticum cluster in Ireland.

## Introduction

Invading populations often experience evolutionary changes and many of these have been attributed to altered selection pressure in the new range (Mooney and Cleland 2001; Lee 2002; Stockwell et al. 2003; Lambrinos 2004; Maron et al. 2004; Barrett et al. 2008; Beckmann et al. 2009). Most frequently, such shifts have become evident in larger sizes and higher growth rates in invasive populations compared to native situations (see Bossdorf et al. 2005 for review). Common explanations for such patterns, for example, include the hypothesis of evolution of increased competitive ability (EICA) in the absence of enemy load in the new range (Blossey and Nötzold 1995; Müller–Schärer et al. 2004). As an alternative, but not necessarily exclusive suggestion, the evolution of increased growth has also been attributed to the absence of competition in a new range as was shown in a multispecies common garden experiment by Blumenthal and Hufbauer (2007). The authors conclude that the biotic release into environments with reduced competition, in particular, in high-resource environments, can favor the evolution of traits related to rapid growth and high net reproductive allocation. The evolution of more competitive phenotypes can be expressed in maximized growth and reproduction (Leger and Rice 2003) as well as in shifts in allocation patterns, for example, from decreased aboveground to increased belowground competition in the new range (Barney et al. 2009). While such differentiation in phenotypes can be quantified at the trait level in common garden attempts, more mechanistic explanations need to refer to hypotheses at the molecular level. The maintenance of high genetic diversity during invasions is a precondition for selection being able to act on (Hedrick 2005). Besides polyploidy, in particular, hybridization can augment genetic novelty and has been suggested to be an important driver promoting evolutionary change in the introduced range (Abbott 1992; Ellstrand and Schierenbeck 2000; Blum et al. 2007; Rieseberg et al. 2007; Prentis et al. 2008).

Invoking hybridization in invasion biology is attractive, since consequences of hybridization, such as fixed heterosis in new allopolyploids, can already have immediate impact on the invasive potential in early stages of plant expansion. Hybridization might also be important in later stages of establishment and spread if adaptive introgression and transgressive segregation aid in the colonization of new environments. An example for recent hybrid speciation is given by *Senecio squalidus* in the British Isles, a recombinant hybrid of *S. aethnensis* and *S. chrysanthemifolius* (James and Abbott 2005). Both parental species were introduced into botanical gardens in Britain in the 18th century, and until now, the hybrid descendant has become widespread throughout the country. Hybrids can exhibit traits that are novel or extreme relative to those of either parental line (Arnold and Hodges 1995) and can differ remarkably in growth rates, phenology, or traits of defense (Vilà et al. 2000). However, hybridization can also cause adaptive trait introgression, through which alleles are transferred from one species to another (Schweitzer et al. 2002). There are several examples that provide evidence for hybrid advantages in fitness-related traits, for example, mediated by increased lifetime fecundity or increased survivorship and higher fruit production for invasive hybrid-derived populations (Campbell et al. 2006; Ridley and Ellstrand 2009). Accordingly, information on demographic characteristics, in particular germination and survival, are important data to assess the species’ susceptibility to novel trait transgression (Hooftman et al. 2005). There is increasing evidence of intrataxon hybridization preceding the evolution of invasiveness (Schierenbeck and Ellstrand 2009). In particular, for ornamental plants or species of horticultural interest, hybridization between cultivars and native counterparts is increasingly drawing the researchers’ attention (Culley and Hardiman 2008; Ross and Auge 2008).

Many of the hypotheses raised above have also been consulted to explain *Rhododendron ponticum* L. invasion in the British Isles. *Rhododendron ponticum* is an Ericaceae shrub species that naturally occurs along the Black Sea coasts of Georgia (Caucasus) and Turkey, as well as in the southern part of the Iberian Peninsula, and was introduced to the British Isles in 1763 (Elton 1958) and used in gardens and estates as an ornamental plant (Dehnen–Schmutz and Williamson 2006). Many *Rhododendron* species from Asia, but also from North America have been introduced to Botanical Gardens in the United Kingdom, mainly for horticultural purposes, and much effort was put into breeding ambitions to make *R*. *ponticum* hardier by natural and artificial selection and by intended hybridization with related species (Dehnen–Schmutz and Williamson 2006). In addition, it was directly brought to habitats with suitable conditions, and naturalization in the wild seems to have occurred all over Britain until the mid of the 19th century (as summarized in Dehnen–Schmutz and Williamson 2006). Flowering starts early in the species’ life cycle, after 10–12 years (Cross 1975), which is a relatively short interval for a woody, long-lived plant and, thus, provides ample opportunity for contemporary evolution. In comparison to their native provenances, invasive Irish populations of *R. ponticum* exhibit both higher germination success and higher growth rates than native ones from Georgia and Spain (Erfmeier and Bruelheide 2005), and these traits have been identified as key factors for establishment and spread in the field (Erfmeier and Bruelheide 2004). Hybridization as a key factor underlying the evolution of invasiveness has repeatedly been suggested for explaining the colonization success of *R. ponticum* in the British Isles (Milne and Abbott 2000; Abbott et al. 2003; Rieseberg et al. 2007). Milne and Abbott (2000) used restriction fragment length polymorphisms of cpDNA and rDNA to study naturalized accessions of *R. ponticum* in the British Isles and detected a mainly Iberian provenance of these invasive occurrences with 99% of Iberian haplotypes. In addition, for both genetic and morphological markers, the authors found evidence of introgression from North American congeneric species of the *Pontica* subsection of the genus (Milne and Abbott 2000). In their study, introgression from North American *R. catawbiense* was remarkable, particularly, in eastern Scotland; therefore, the authors suggest that hybridization had induced cold tolerance that enabled southern Iberian *R. ponticum* provenances to spread into northern climates. However, in their study, the comparison of British and North American taxa included small sample sizes of the North American *R. catawbiense* and *R. maximum*. In addition, marker systems applied referred to plastid DNA that is maternally inherited and, thus, does not reflect effects of recombination. Thus, the question as to which mechanism has contributed to this differentiation encountered among the provenances remains a fundamental issue for explaining *R. ponticum* invasion. In particular, it is still unresolved whether hybridization might have contributed to this differentiation.

The present study aims to address the question of hybridization with North American *R. catawbiense* and/or *R. maximum* for explaining the invasive spread of *R. ponticum* in Ireland on the basis of both molecular marker profiles and quantitative traits of germination success and further ecological and morphological traits. We carried out a field sampling campaign including six populations each from European invasive and native *R. ponticum* in Ireland, Georgia, and Spain, respectively, and six populations each from North American *R. catawbiense* and *R. maximum* to gain seed and leaf material according to a common design and to test for similarities in genetic and quantitative markers. In particular, our aims were to (1) identify patterns of phenotypic trait variation and differentiation among populations within native and introduced *Rhododendron* taxa and (2) assess the relative contribution of hybridization to explaining the encountered patterns. Against this background, we discuss the evolutionary dimension of increased invasiveness of *R. ponticum* in Ireland.

## Material and Methods

### Study objects

All species and origins included in our study belong to the genus *Rhododendron*, section *Pontica*, within the Ericaceae family (Milne 2004). Areas of primary distribution of most *Rhododendron* species are SW China and the Himalayas. However *R*. *ponticum* distribution is confined to southwestern Eurasia, whereas *R*. *maximum* and *R*. *catawbiense* are native to southeastern North America. Natural occurrences of *R. ponticum* can be found mostly in forests on acid substrate of the eastern Balkan Peninsula, along the Black Sea Coast, and in riparian forests on the Iberian Peninsula, however, populations being in decline in that Iberian part of the native range (Mejías et al. 2007). In its invasive range, in the British Isles, *R. ponticum* has invaded forests, heathlands and bogs, and requires enormous control efforts and eradication attempts (Dehnen–Schmutz et al. 2004). *Rhododendron catawbiense* is a typical species of open woodlands and scrub at higher elevation in the Appalachian Mountains, USA. In contrast, *R. maximum* is preferably found in moist and wet forests in eastern North America. Although there is very little agreement between geographic location and phylogenetic position within the subsection *Pontica* (Milne 2004), there are nonetheless similarities among these species with respect to morphological traits. Leaf characteristics, for example, leaf apex and leaf base, are quite similar for *R. ponticum* and *R. maximum*. In contrast, *R. ponticum* and *R. catawbiense* are much more alike in terms of leaf coloring and glabrous twigs (Gleason and Cronquist 1963; Tutin et al. 1972; Clapham et al. 1987; Weakley 2000).

In accordance with the disjunctive distribution, *R. ponticum* in Turkey and in Georgia (Caucasus) is assigned to ssp. *ponticum* (Tutin et al. 1972; Davis 1978); whereas occurrences from southern Spain and Portugal are taxonomically addressed as ssp. *baeticum* (Boiss. & Reuter) Hand.-Mazz. (Tutin et al. 1972; Davis 1978; Clapham et al. 1987; Castroviejo et al. 1993). The taxonomic distinction between the two subspecies is clearly apparent in leaf shape differences: leaves of ssp. *ponticum* have a length of 12-18-(25) cm and are 2.5–3.5 times as long as wide, while leaves of ssp. *baeticum* are shorter with 6-12-(16) cm and have a larger length–width ratio of 3–5 than ssp. *ponticum*.

### Material and sampling design

For the experiments and the molecular analyses, we used seed and leaf material from all three species; for *R. ponticum*, we sampled native subspecies from Georgia (ssp. *ponticum*) and from southern Spain (ssp. *baeticum*) as well as invasive provenances from Ireland. For each species and provenance (henceforth called taxa), six populations in the respective area of distribution were chosen randomly with the intention to cover maximum variation. We only included *Rhododendron* stands within forests at sites with a northern aspect and a slope of 10° to 20° to ensure comparability among the countries. Seeds from native *R. ponticum* were collected in Georgia (GEO) in August, from native *R. ponticum* in Spain (ESP) in October, from invasive *R. ponticum* in Ireland (IRE) in September, from native *R. catawbiense* (CAM) and *R. maximum* (MAM) in the Appalachian Mountains in North America in October, all in 2001. The exact locations of all 30 populations (five taxa with six populations each) sampled are provided in Table 1. Further details on the selection mode are described in Erfmeier and Bruelheide (2004).

**Table 1:** Overview on populations sampled and location characteristics of the sites

Taxon	Country	Population	Location	Elevation [m.a.s.l.]	Latitude	Longitude
GEO	Georgia	A	Banis-Khevi	980	41°53′	E 043°21′
GEO	Georgia	B	Keda-Akutsa	500	41°35′	E 041°57′
GEO	Georgia	C	Dandalo	910	41°38′	E 042°07′
GEO	Georgia	D	Botanical Garden, Batumi	85	41°41′	E 041°43′
GEO	Georgia	E	Djarnali	175	41°33′	E 041°36′
GEO	Georgia	F	Mtirala	960	41°39′	E 041°47′
ESP	Spain	G	Garganta de Puerto Oscuro	790	36°30′	W 005°37′
ESP	Spain	H	Garganta de Passada Llana	760	36°30′	W 005°35′
ESP	Spain	I	Arroyo del Montero	660	36°29′	W 005°35′
ESP	Spain	K	Garganta de Enmedio	445	36°32′	W 005°38′
ESP	Spain	L	Llanos del Juncal	740	36°06′	W 005°32′
ESP	Spain	M	Rio de la Miel	430	36°06′	W 005°31′
IRE	Ireland	N	National Park Killarney, Torc Mnts.	60	52°00′	W 009°30′
IRE	Ireland	O	National Park Killarney, Ladies View	35	51°58′	W 009°35′
IRE	Ireland	P	Glengariff	35	51°45′	W 009°33′
IRE	Ireland	Q	Galtee Mnts.	180	52°22′	W 007°58′
IRE	Ireland	R	Knockmealdown Mnts.	220	52°15′	W 007°57′
IRE	Ireland	S	Greenan, Wicklow Mnts.	120	52°55′	W 006°18′
MAM	USA	A	Two Chimneys, Great Smokey Mnts. National Park	1075	35°38′	W 083°28′
MAM	USA	B	The Sinks, Great Smokey Mnts. National Park	545	35°40′	W 083°39′
MAM	USA	C	Mingus Mill, Great Smokey Mnts. National Park	605	35°30′	W 083°19′
MAM	USA	D	Saunakee Village Viewpoint, Blue Ridge Parkway National Park	1365	35°25′	W 083°02′
MAM	USA	E	Linville Falls, Blue Ridge Parkway National Park	1035	35°56′	W 081°55′
MAM	USA	F	Green Know, Blue Ridge Parkway National Park	1390	35°42′	W 082°14′
CAM	USA	A	Two Chimneys, Great Smokey Mnts. National Park	1120	35°38′	W 083°28′
CAM	USA	B	Mt. Sterling, Great Smokey Mnts. National Park	1721	35°42′	W 083°06′
CAM	USA	C	Waterrock Knob, Blue Ridge Parkway National Park	1815	35°27′	W 083°08′
CAM	USA	D	Richland Balsam Summit, Blue Ridge Parkway National Park	1846	35°21′	W 082°59′
CAM	USA	E	Craggy Gardens, Blue Ridge Parkway National Park	1725	35°41′	W 082°22′
CAM	USA	F	Grandfather Mountain, Blue Ridge Parkway	1615	36°05′	W 081°49′

GEO = *Rhododendron ponticum*, Georgia; ESP = *R. ponticum*, Spain; IRE = *R. ponticum*, Ireland; MAM = *R. maximum*, North America; CAM = *R. catawbiense*, North America.

Within each population, seeds were collected randomly from at least 20 fruiting individuals with a minimum distance of 5 m. Only ripe racemes were harvested. Afterwards released seeds were thoroughly mixed within each population's sample and stored in a dry, dark place at ambient temperatures of 10°C until further use. Accordingly, we collected cuttings in each population for frost hardiness studies on rerooted branches. For sampling and cultivation details, see Erfmeier and Bruelheide (2005). Leaf material for molecular genetic analyses was collected in each population according to a systematic sampling scheme on the basis of a 16 × 16 m grid with a mesh width of 4 m. Samples from the four central individuals each were included in the molecular analyses. Leaf material of all *R. ponticum* populations was collected in winter 1999/2000; *R. maximum* and *R. catawbiense* populations were sampled in autumn 2001. All leaves were dried and stored in silica gel prior to analysis.

### Genetic analyses

From silica-dried leaves of a total of 120 individuals (i.e., four individuals per population with six populations per taxon), DNA was isolated with DNA Puregene cell and tissue Kit (Gentra Systems, Minneapolis, MN, USA) using 30–40 mg of dry leaf material. Genotyping was performed using the Amplified Fragment Length Polymorphism (AFLP) technique according to Vos et al. (1995) with modifications as described in Erfmeier and Bruelheide (2011). For each leaf sample, a total of three amplification procedures were run, including each DNA of two separate extractions. Fluorescently labelled PCR products were analyzed on an automated gel sequencer ABI PRISM ® 3100 (Applied Biosystems, Foster City, CA, USA) to infer sample specific fragment patterns. Fragments were analyzed with the software GEN SCANNER as described in Müller et al. (2005). The three replicates per individual were combined, compared, and translated into a 0-1 matrix. For recognition of presence and absence of peaks, in the three parallel analyses, a peak was considered as being present if the occurrence of peaks was provided in two or three of the replicates of this individual. All other peaks were regarded as error.

In order to identify traces of putative introgression, private and common markers were counted for all combinations of taxa. Assuming that hybrids should contain diagnostic alleles from their parent taxa, we counted diagnostic markers of the four potential parent taxa and tested for presence of these specific peaks in the Irish taxon.

### Germination experiment

Seeds from each of the six populations per taxon were subjected to three different temperature regimes of 9/19°C, 16/26°C, and 23/33°C (night/day) with a thermo- and photoperiod of 16-h day length. Each 20 seeds were placed in petri dishes on 70 g of sterilized sand (105°C for 24 h), being covered with paper discs (Schleicher & Schuell GmbH, Dassel, Germany; diameter 90 mm), and kept constantly moist with deionized water. Petri dishes were regularly watered and sprayed with 50% ethanol solution twice a week to suppress mildew infection.

Each population was replicated three times at each temperature level, yielding 270 petri dishes being randomly positioned in controlled environment cabinets (Heraeus Vötsch, Vötsch Industrietechnik GmbH, Frommern, Germany). The environment cabinets were equipped with white light providing 120–150 µE/m^2^/s on average. Germination success was monitored every third day in the beginning and in total 10 times during the whole experimental period of 70 days. Seeds were considered to have germinated when the first radicle had emerged and were transferred at the cotyledon stage to pots (7 × 7 × 8 cm^3^) with 70%:30% sand–peat substrate for further cultivation.

### Growth experiment

Seedlings for the growth experiment were acquired from the germination experiment and from additional seeds sown directly into pots. Seedlings from both cultivation attempts had about 3–4 weeks after germination for establishing in their pots before being exposed to growth treatments. In controlled greenhouse cabinets, the plants were subjected to a daily alternating temperature regime of either 9/19°C, 16/26°C, and 23/33°C and a thermo- and photoperiod of 8/16 (night/day) hours. Each cabinet was split into two layers of different light regimes: with the upper layer having a light treatment of 400 mE/m^2^/s and the layer underneath experiencing reduced light availability of 40 mE/m^2^/s. In addition, plants were subjected to two soil water level treatments of 25% and 15% (of dry weight). The combinations of these experimental settings resulted in a total of 12 experimental environments, to which all of the 30 populations available (each six populations by taxon) were assigned to. A total set of 360 individuals were randomly placed (within treatments) in the cabinets.

Water levels of the pots were assessed gravimetrically and readjusted every second day. At 3-week intervals, the pots were fertilized with a 0.25‰ NPK fertilizer (Flory 1, EUFLOR GmbH, München, Germany); after 6 weeks, fertilizer concentration was doubled for all pots to account for the seedling's increase in biomass. The seedling growth experiment ran for about 12 weeks. We studied variables of growth (increase in total plant height, number of leaves), variables of allocation (above- and belowground biomass, shoot–root ratio) and traits of leaf morphology (length and width of the largest leaf). Monitoring of size- and growth-related variables occurred once in the beginning and subsequently every 3 weeks. Biomass data were assessed at the end of the experiment by determining root and shoot dry weight. For variables of increase, we calculated relative growth rates (RGRs) following Hunt (1989).

### Frost hardiness

Frost hardiness was determined on cuttings that had been harvested in autumn 2001 of all origins and taxa and that had been cultivated in the greenhouse at temperatures of 12/17°C (night/day) during the winter. For frost hardiness testing, we selected individuals from all populations and taxa at random and kept them for 7 d in a refrigerator of 4°C prior to the experiment for acclimation and hardening. From these individuals, we sampled leaves that still corresponded to plant material that had developed in the field. Exposure to frost treatments of these leaves started in April 2002.

Frost hardiness was quantified using the electrolyte leakage method according to Murray et al. (1989), which relates increasing tissue damage caused by frost to increasing rates of electrolyte loss. The experiment was carried out with 11 2 × 1 cm^2^ leaf rectangles from each individual, each one being assigned to one of 11 temperature levels applied, thus representing connected samples that allow for calculating nonlinear regressions across all temperature levels for every individual (Kathke and Bruelheide 2011). The five taxa with each six populations were represented by one individual each, yielding a total of 330 leaf samples. Leaf samples were subjected to freezing temperatures in a freezing chamber of –6°C, –9°C, –12°C, –15°C, –18°C, –21°C, –24°C, –27°C, and –30°C, respectively, or to a nonfreezing control temperature (+4°C). We chose a fine resolution of temperature treatments down to –30°C to reveal taxa differentiation more precisely and to grasp the full range of cold hardiness described for *R. ponticum* in literature (Sakai et al. 1986). Each temperature level lasted for 2 h with a cooling period of 1 h between levels. In addition, one leaf sample per individual was subjected to a liquid N_2_ (–196°C) treatment for 2 h to obtain maximum frost damage. After experimental frost exposure, leaf samples were put into tubes with 10-mL 3% iso-propanol solution. The electrical conductivity of the solution was measured (LF 2000, WTW) immediately after immersing the disk (C0) and subsequently repeated after 5 h, 19 h, 43 h, and 92 h (Ct). At the end of the measurement series, the solution with the leaf disk was boiled for 20 min, thus releasing all electrolytes from the cells, and final conductivity was measured as individual maximum reference (C_max_).

Each measurement was expressed as relative conductivity (RC) according to Murray et al. (1989) to account for variation in total electrolyte content. All RC values of one time series were fitted to a one-parameter nonlinear regression (proc nlin SAS 9.1, SAS Institute Inc., 2000):

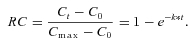


The resulting parameter *k* indicates the rate of electrolyte leakage and can be used for further analyses as a measure of frost hardiness.

### Statistical analysis

The presence/absence matrix of DNA fragments was analyzed with a cluster analysis based on unweighted pair group method with arithmetic average (UPGMA).

Data on growth, biomass, and on leaf morphology in the growth experiment were analyzed with general linear models (GLMs) for unbalanced data (type III sum of squares, proc glm). Prior to analysis, all growth and germination data were rank transformed as the majority of data lacked normal distribution (proc univariate; for appropriateness of rank transformation, see Brunner and Puri 2001; Quinn and Keough 2002). Maximum germination success was tested for effects of the taxon and the temperature applied, both considered fixed factors and with populations as random factor nested within taxon. Effects of taxon and temperature on germination velocity were tested in repeated measures analysis of variance (ANOVA; repeated statement in proc glm) with “time” as additional factor. Since our data did not satisfy the assumption of sphericity, numerator and denominator degrees of freedom were adjusted before determining significance levels according to Greenhouse–Geisser. In all growth analyses, we tested for main and interaction effects of taxon, temperature (both in germination and growth analyses), light and water (in growth analyses) as fixed factors and with populations as random factor nested in taxon (pop(tax)). Post-hoc tests were realized with Ryan–Einot–Gabriel–Welsh (REGWQ) multiple range tests.

For analysis of frost hardiness, we applied the same GLM to test for taxon, temperature, and their interaction effects on the rate of electrolyte leakage *k*. In addition, all variables were also compared using contrasts of the control temperature of +4°C versus all other temperatures of the freezing treatment separately by taxon (contrast and estimate statements, proc glm, SAS 9.1; SAS Institute Inc. 2000). The temperature at which 50% of the maximum *k* value occurred (*LT*_50_) was calculated by fitting a four-parameter nonlinear regression to the *k* values (Sigma Plot 9.0, Systat Software 2004) using the following equation:

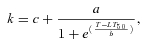
with *k* the rate of electrolyte leakage; *T* the temperature to which the leaf sample was exposed; and *a, b, c*, and *LT*_50_ as regression parameters. *LT*_50_ was calculated for each taxon across all populations and temperature replicates.

All phenotypic data were summarized and analyzed by discriminant analysis in order to assign invasive and putative hybrid Irish phenotypes to one or more of the considered parental taxa. We performed a stepwise discriminant analysis (proc stepdisc) to select a subset of significant quantitative variables to discriminate between the four native Georgian, Spanish, and North American taxa. We applied a forward selection procedure, using all variables of growth, morphology, allocation, germination, and frost sensitivity, which consecutively entered the model according to the significance level of an *F*-test. Forward selection began with no variables in the model and stopped when no further variable could be added at the significance level of *P* = 0.05. Based on the final set of discriminating variables, we calculated the error probability of incorrect assignment for each of the four classified taxa by means of a discriminant procedure (proc discrim). In the following, the resulting discriminant function was applied to the Irish dataset to assign these invasive populations to either one of the European Georgian or Spanish *R. ponticum* or to the North American congeneric species *R. catawbiense* or *R. maximum*.

Illustration of taxon separation was performed with linear discriminant analysis (LDA) using R 2.10.1 based on the set of discriminating variables only. Multivariate observations were classified with lda (Mass package) and projected onto the first two linear discriminants.

## Results

### Genetic relationships between *Rhododendron* taxa

AFLP scores generated 478 polymorphic markers from three primer pairs across all 120 samples analyzed. All individuals had unique fragment combinations, thus, displayed discrete genotypes. Private alleles were rarely encountered: 4.4% of all alleles (i.e., 21 alleles) were present in all individuals, further 19.5% were shared by all populations (i.e., 93 alleles), and 50% were in common in all five taxa studied (i.e., 239 alleles; data not shown). A total of 24 alleles were found to be exclusive, that is, taxon-diagnostic, with 10 diagnostic alleles for *R. catawbiense*, six alleles each for *R. maximum* and Georgian *R. ponticum*, and each one diagnostic allele for Spanish and Irish *R. ponticum* (see Appendix S1). Inter-taxa comparisons between North American *Rhododendron* taxa with European *R. ponticum* taxa displayed only few, exclusively common alleles: while *R. catawbiense* exclusively shared one allele with Irish *R. ponticum*, the number of exclusively shared alleles between *R. maximum* and Irish *R. ponticum* was three alleles. For the North American taxa, the number of diagnostic markers shared with Georgian and Spanish *R. ponticum* was at a similar height, with five and three alleles, respectively, for *R. catawbiense* and with five and one alleles, respectively, for *R. maximum*.

**Figure 1:**
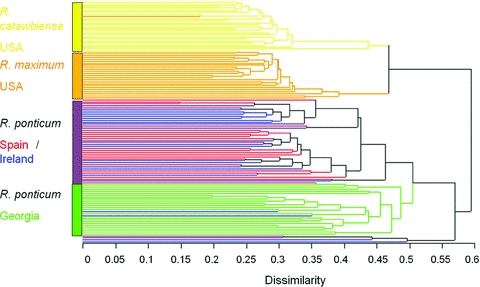
Cluster analyses based on unweighted pair group method with arithmetic average (UPGMA) of AFLP profiles of 24 genotypes each from *Rhododendron catawbiense* (North America), *R. maximum* (North America), invasive Irish *R. ponticum*, native Spanish *R. ponticum*, and native Georgian *R. ponticum*.

The level of dissimilarity among individuals was low, ranging from 0.148 to 0.748. UPGMA analyses revealed a clear distinction of the three *Rhododendron* species (Fig. 1), with the major separation between a North American cluster including *R. maximum* and *R. catawbiense* and a European *R. ponticum* cluster. Within the North American cluster, both species were clearly distinguished with only one single *R. maximum* individual clustering among *R. catawbiense*. Within *R. ponticum*, the Georgian taxa clustered separately from the Iberian and Irish provenances. In contrast, individuals of the Spanish and the Irish provenances were completely intermixed and clustered together.

### Germination characteristics

**Figure 2:**
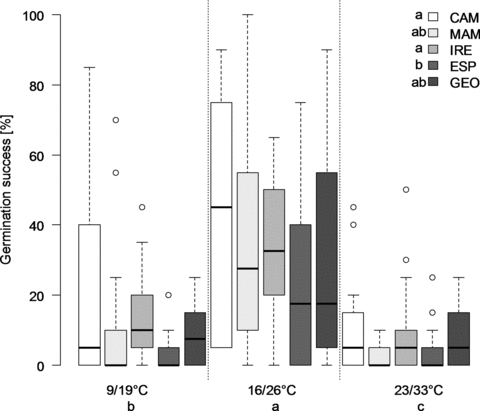
Germination success of *Rhododendron* taxa's seeds across three temperature regimes (*n* = 270). Medians, quartiles, minimum, and maximum refer to six populations with three replicates each per temperature (*n* = 18). Different letters indicate significant differences according to the REGWQ-test. CAM = North American *R. catawbiense*; MAM = North American *R. maximum*; IRE = invasive Irish *R. ponticum*; ESP = native Spanish *R. ponticum*; GEO = native Georgian *R. ponticum*. For statistical details referring to rank transformed data, see Table 2.

Maximum germination success differed clearly between the *Rhododendron* taxa tested after 10 weeks of temperature treatments (Table 2A; Fig. 2). We found the highest values for North American *R. catawbiense* and invasive Irish *R. ponticum*, which differed significantly from native Spanish *R. ponticum*. Maximum germination of native Georgian *R. ponticum* and North American *R. maximum* was intermediate and neither differed significantly from invasive Irish nor from native Spanish *R. ponticum*. Germination success was significantly higher at the intermediate temperature level compared to the lowest and the highest temperature levels.

**Table 2:** GLM results for germination tests of *Rhododendron* taxa: (A) Maximum germination success after 10 weeks; Germination data were rank transformed prior to analysis. (B) Cumulative germination success expressed by repeated measures ANOVA with factor time. The mixed model was performed with populations as random factor nested within taxon and three replicates each per temperature level (*n* = 270). *P* GG adj = *P* values for Greenhouse–Geisser adjustment. Bold numbers indicate significant effects

(A)						
Source of variation	*df*	Type III SS	MS	*F*	*P*	
Taxon	4	78136	19534	3.93	**0.013**	
Error (pop(taxon)).	25	124429	4974.3			
Temperature	2	378100	189050	45.65	**<0.001**	
Temperature × taxon	8	24615	3076.9	0.74	0.654	
Pop(taxon)	25	124365	4974.6	1.2	0.239	
Error	230	952522	4141.4			

(B)						
Source of variation	*df*	Type III SS	MS	*F*	*P*	*p*_GGadj_
Time	10	58595.2	5859.5	135.0	**<0.001**	**<0.001**
Time × temperature	20	30389.1	1519.5	35.0	**<0.001**	**<0.001**
Time × taxon	40	6118.3	153.0	3.5	**<0.001**	**<0.001**
Time × temperature × taxon	80	3188.6	39.9	0.9	0.681	0.543
Time × pop(taxon)	250	12809.7	51.2	1.2	**0.034**	0.203
Error(time)	2300	99813.1	43.4			
Greenhouse–Geisser–Epsilon	0.185					

We found significant taxon effects for germination velocity, which was indicated by significant time × taxon interactions: seeds from *R. catawbiense* and Irish *R. ponticum* initially responded much faster to the temperature treatments than seeds from all other taxa (Table 2B). This pattern was consistent for all temperature levels studied, as displayed by nonsignificant time × taxon × temperature interaction effects.

### Seedling characteristics

**Figure 3:**
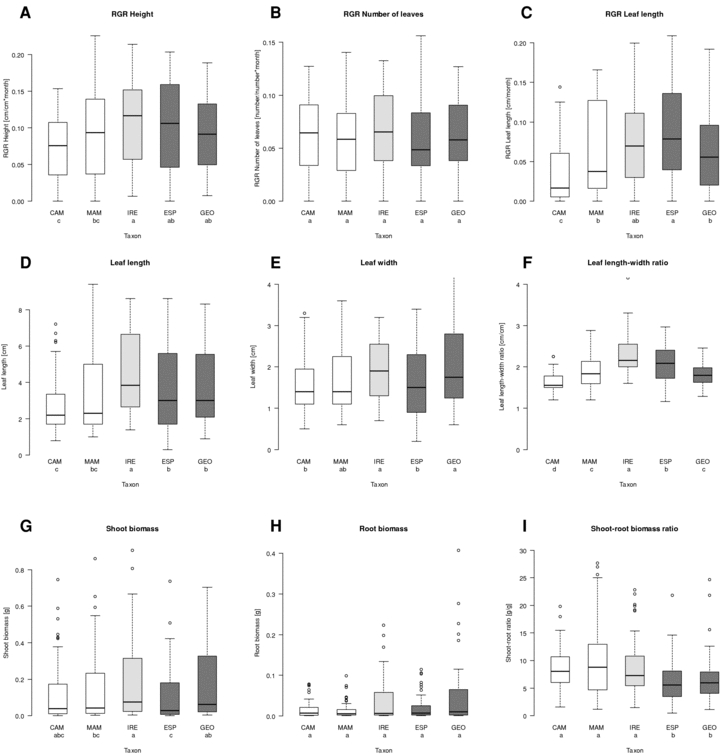
Taxon effects on traits of growth (a–c), leaf morphology (d–f), and biomass allocation (g–i) of *R. ponticum* seedlings across three temperature, two light, and two water regimes. (a) RGR in height, (b) RGR in number of leaves, (c) RGR in leaf length, (d) Leaf length, (e) Leaf width, (f) Leaf length-width ratio, (g) Shoot dry biomass, (h) Root dry biomass, (i) Shoot-root biomass ratio. Medians, quartiles, minimum, and maximum refer to six populations each as replicates across 12 treatments (*n* = 72). Different letters indicate significant differences according to the REGWQ-test. CAM = North American *R. catawbiense*; MAM = North American *R. maximum*; IRE = invasive Irish *R. ponticum*; ESP = native Spanish *R. ponticum*; GEO = native Georgian *R. ponticum*. For statistical details, see Table 3.

After 12 weeks of the experiment, all variables of growth and performance differed significantly between the five taxa (Table 3). Growth traits most often showed highest magnitude for the invasive Irish *R. ponticum* taxon, which had the largest relative growth rates in height, in number of leaves as well as in leaf length, and in width (Fig. 3A–C; Table 3). Relative growth rates in height and leaf width did not differ significantly among all *R. ponticum* taxa (Fig. 3A; Table 3), and for RGR in leaf length (Fig. 3C), both the Spanish and the Irish taxon displayed highest growth rates. Apart from RGR in number of leaves (Fig. 3B), we found a clear dissimilarity in all growth traits for invasive Irish *R. ponticum* and the American taxon *R. catawbiense*; the latter consistently displayed significantly lower growth rates than the invasive Irish congeneric *R. ponticum*.

**Table 3:** GLM analysis for traits of (1) growth, (2) leaf morphology, and (3) biomass of *R. ponticum* seedlings after 12 weeks. All variables were rank transformed prior to analysis. Listed are effects of taxon, temperature treatments, light conditions, water conditions, and their interactions as fixed effects. ANOVA was performed with populations as random factor nested within taxon (*n* = 360)

Source of variation	RGR Height			RGR no. of leaves			RGR leaf length			RGR leaf width			Leaf length			Leaf width		
						
	*df*	*F*	*P*	*df*	*F*	*P*	*df*	*F*	*P*	*df*	*F*	*P*	*df*	*F*	*P*	*df*	*F*	*P*
Taxon	4	7.48	***	4	3.84	*	4	18	***	4	8.73	***	4	13.8	***	4	6.75	***
Error	25.8			25.8			25.7			25.9			25.6			25.6		
Temperature	2	0.47		2	15.98	***	2	102	***	2	86.3	***	2	77.6	***	2	69.8	***
Taxon × temperature	8	1.83		8	2.22		8	2.8	**	8	2.13	*	8	0.96		8	1.15	
Light	1	987	***	1	759.3	***	1	759	***	1	705	***	1	737	***	1	743	***
Taxon × light	4	4.6	**	4	0.75		4	0.2		4	0.43		4	2.23		4	2.36	
Temperature × light	2	0.37		2	5.39	**	2	23.2	***	2	11.1	***	2	19.8	***	2	18.5	***
Taxon × temperature × light	8	1.02		8	1.64		8	4.45	***	8	3.23	**	8	1.18		8	1.82	
Water	1	1.95		1	1.24		1	0.34		1	3.53		1	0.02		1	1.18	
Taxon × water	4	0.54		4	0.97		4	0.8		4	0.38		4	0.6		4	0.23	
Temperature × water	2	0.56		2	0.53		2	0.97		2	0.89		2	0.34		2	1.68	
Taxon × temperature × water	8	0.76		8	0.76		8	1.76		8	1.48		8	0.77		8	1.03	
Light × water	1	0.01		1	0.41		1	0.16		1	0.35		1	0.05		1	0.14	
Taxon × light × water	4	0.25		4	0.16		4	2.06		4	3.57	**	4	0.67		4	0.58	
Temperature × light × water	2	2.54		2	0.01		2	2		2	2.7		2	1.21		2	1.32	
Taxon × temperature × light × water	8	0.48		8	0.37		8	1.27		8	1.03		8	0.42		8	0.55	
Pop(taxon)	25	1.94	**	25	1.91	**	25	1.96	**	25	1.64	*	25	2.43	***	25	2.34	***
Error: MS(error)		264			264			264			264			264			264	

Source of variation	Leaf length–width ratio			SLA			Shoot biomass			Root biomass			Shoot–root ratio					
								
	*df*	*F*	*P*	*df*	*F*	*P*	*df*	*F*	*P*	*df*	*F*	*P*	*df*	*F*	*P*			
Taxon	4	32.8	***	4	9.61	***	4	6.42	**	4	3.45	*	4	10	***			
Error		25.6			27.9			25.8			25.9			27.3				
Temperature	2	37.3	***	2	1.24		2	11.2	***	2	8.87	***	2	0.68				
Taxon × temperature	8	1.26		8	1		8	0.89		8	1.95		8	4.78	***			
Light	1	190	***	1	120.5	***	1	877	***	1	721	***	1	25.2	***			
Taxon × light	4	6.05	***	4	3.76	**	4	1.04		4	5.98	***	4	11	***			
Temperature × light	2	6.82	**	2	19.17	***	2	4.63	*	2	2.9		2	2.38				
Taxon × temperature × light	8	1.42		8	1.49		8	1.28		8	0.5		8	1.96				
Water	1	3.62		1	2.61		1	2.44		1	0.02		1	2.76				
Taxon × water	4	2.05		4	0.26		4	0.61		4	0.2		4	1.57				
Temperature × water	2	2.5		2	0.5		2	0.18		2	1.96		2	0.93				
Taxon × temperature × water	8	1.09		8	0.74		8	0.12		8	0.75		8	1.01				
Light × water	1	0		1	0.03		1	0		1	0.25		1	0.01				
Taxon × light × water	4	0.57		4	0.27		4	2.84	*	4	3.16	*	4	0.1				
Temperature × light × water	2	1.48		2	2.2		2	0.95		2	0.18		2	1.06				
Taxon × temperature × light × water	8	1.27		8	1.28		8	0.42		8	1.09		8	0.79				
Pop(taxon)	25	2.42	***	25	1.2		25	2.35	***	25	2.5	***	25	1.05				
Error: MS(error)		264			255			262			254			254				

Leaf length and leaf-length-width ratio (Fig. 3D and F) were significantly larger for Irish *R. ponticum* than for all other taxa. Leaf width (Fig. 3E) and SLA (Table 3) did not differ between the invasive Irish taxon and native *R. ponticum* from Georgia and *R. maximum*. American *R. catawbiense* differed significantly in all leaf traits analyzed from invasive Irish *R. ponticum* (Table 3).

While the post-hoc test on root biomass revealed no significant differences between taxa (Fig. 3H), highest shoot biomass was encountered for invasive Irish and native Georgian *R. ponticum* in common with *R. catawbiense* (Fig. 3G); the Irish taxon displayed significantly higher aboveground biomass than Spanish *R. ponticum* and *R. maximum*. The shoot–root biomass ratio (Fig. 3I) was highest for the American *Rhododendron* taxa together with invasive Irish *R. ponticum*. All three taxa differed significantly from native *R. ponticum* taxa.

For most of the variables, we found some interaction effects indicating that the different taxa responded differently to different temperature and light environments, respectively (Table 3). RGR in number of leaves and shoot–root biomass ratio, for example, showed differential increase at warmer temperatures (see Appendix S2): in particular, North American *R. catawbiense* and *R. maximum* and the invasive Irish *R. ponticum* were able to increase growth and shoot–root ratios at warmer temperatures compared to native *R. ponticum*. Taxa × light interactions for RGR in height indicate that the invasive Irish *R. ponticum* and its native Spanish conspecific and *R. maximum*, in particular, seemed to profit from higher light conditions (Table 3; Appendix S2).

### Frost hardiness

**Figure 4:**
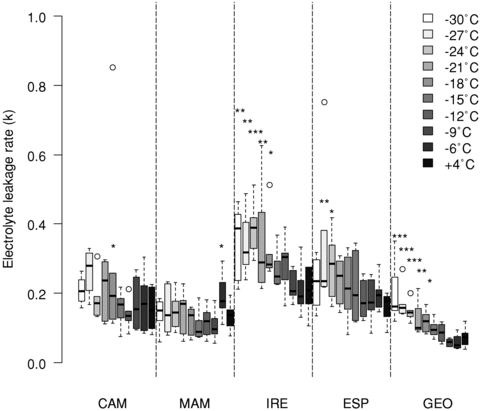
Electrolyte leakage rate *k* for the *Rhododendron* taxa at 10 different temperature levels. CAM = North American *R. catawbiense*; MAM = North American *R. maximum*; IRE = invasive Irish *R. ponticum*; ESP = native Spanish *R. ponticum*; GEO = native Georgian *R. ponticum*. Stars indicate significant differences according to contrasts between the control temperature of +4°C versus all other temperatures within each taxon. *P < 0.05, **P <0.01, ***P < 0.001.

**Figure 5:**
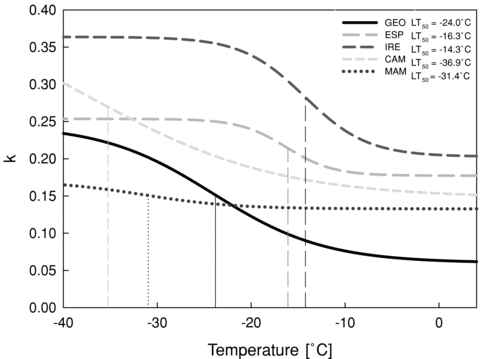
Estimation of the *LT*_50_ values for all *Rhododendron* taxa referring to *k* values and the corresponding temperature levels of the freezing treatment. Regression follows a four-parameter sigmoid function *k* = *c*+*a*/(1+exp(–(*T*–*LT*_50_)/*b*)), where *T* is the temperature to which the leaves were exposed and *a, b, c*, and *LT*_50_ are regression parameters. The regression was applied to pooled data per taxon (*n* = 60). CAM = North American *R. catawbiense*; MAM = North American *R. maximum*; IRE = invasive Irish *R. ponticum*; ESP = native Spanish *R. ponticum*; GEO = native Georgian *R. ponticum*. All regressions were significant (*P* < 0.001) with the exception of MAM. Vertical lines mark the *LT*_50_ values as position of the inflection point on the x-axis.

Tested across all taxa and compared to the control level, the electrolyte leakage rate *k* was significantly larger at all temperature levels of –18°C or below this value, suggesting general frost hardiness down to at least –15°C (see Appendix S3). The invasive Irish *R. ponticum* displayed the highest rates of frost damage and differed significantly from all other taxa (Fig. 4; Table 4). Damage was significantly different from the 4°C control level for Irish and Georgian samples at –18°C and for Spanish samples at –24°C, while for the North American taxa, we found no significant differences compared to the control. Frost hardiness expressed as *LT*_50_ values following nonlinear four-parameter regression was highest for North American *R. catawbiense* and *R. maximum* with *LT*_50_ values of –36.9°C and –31.4°C, respectively (Fig. 5). Among the *R. ponticum* origins, frost hardiness decreased from native Georgian (–24.0°C) to native Spanish (–16.3°C) and invasive Irish (−14.3°C) populations.

**Table 4:** Summary of GLM analysis for frost sensitivity (rate of electrolyte leakage *k*) of *Rhododendron* taxa. Listed are effects of taxon, temperature treatments, and their interactions as fixed effects. ANOVA was performed with populations as random factor nested within country (*n* = 330). Bold numbers indicate significant effects

Source of variation	*df*	Type III SS	MS	*F*	*P*
Taxon	4	1.237446	0.309361	20	**<0.001**
Error (pop(taxon))	25	0.386344	0.015454		
Temperature	9	0.409223	0.045469	8.04	**<0.001**
Taxon × temperature	36	0.267416	0.007428	1.31	0.121
Pop (taxon)	25	0.386344	0.015454	2.73	**<0.001**
Error	225	1.272515	0.005656		

### Assignment of invasive populations

**Figure 6:**
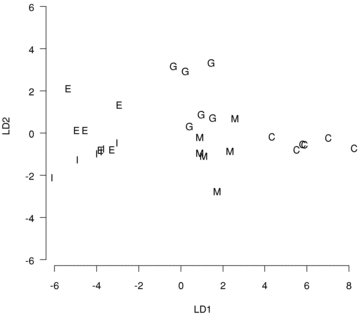
Ordination plot of the first and second linear discriminant functions of the six populations per *Rhododendron* taxon based on the discriminating variables RGR leaf length, leaf length–width ratio, root biomass, and shoot–root ratio. Proportion of explained variance for axis LD1 = 87.76%, LD2 = 6.72%. C = North American *R. catawbiense*; M = North American *R. maximum*; I = invasive Irish *R. ponticum*; E = native Spanish *R. ponticum*; G = native Georgian *R. ponticum*.

Stepwise discriminant analysis revealed the variables RGR leaf length (*F* = 28.09, *P* < 0.001), leaf length–width ratio (*F* = 11.80, *P* < 0.001), root biomass (*F* = 9.14, *P* < 0.001), and shoot–root ratio (*F* = 4.74, *P* = 0.015) to discriminate significantly among the native *Rhododendron* taxa. The overall probability of misclassification was 8.3% (Table 5). Only two of the six native Georgian *R. ponticum* populations were misclassified as *R. maximum*; for all other populations of taxa, posterior probability of membership in the correct taxon was 100% (Table 5). Application of this discriminant function to the Irish dataset revealed 100% classification of the invasive Irish populations to the native Spanish congeneric ones (Table 5). Invasive Irish *R. ponticum* differed considerably from both North American taxa as well as from Georgian *R. ponticum*, and displayed closest relationship to the Spanish *R. ponticum* (Fig. 6).

**Table 5:** Classification summary following linear discriminant analysis for calibration of the native taxa CAM, MAM, ESP and GEO, and the test taxon IRE. Number of observations and percent classified into taxon

From tax	CAM	MAM	ESP	GEO	Total
CAM	6	0	0	0	6
	100%	0	0	0	100%
MAM	0	6	0	0	6
	0	100%	0	0	100%
ESP	0	0	6	0	6
	0	0	100%	0	100%
GEO	0	2	0	4	6
	0	33.3%	0	66.7%	100%
Total	6	8	6	4	24
	25	33.3	25	16.7	1.0
Priors	0.25	0.25	0.25	0.25	
Error counts estimates for taxon		0.083			

From tax	CAM	MAM	ESP	GEO	Total
IRE	0	0	6	0	6
	0	0	100%	0	100%

CAM = North American *Rhododendron catawbiense*; MAM = North American *R. maximum*; IRE = invasive Irish *R. ponticum*; ESP = native Spanish *R. ponticum*; GEO = native Georgian *R. ponticum*.

## Discussion

In the present study, neither ecological nor morphological traits bore a general resemblance of Irish *R. ponticum* with North American *R. catawbiense* or *R. maximum* as reflected, in summary, in the discriminant analysis. Consistently, the AFLP data displayed a clear distinction between all *R. ponticum* taxa on the one hand and the two North American species on the other hand. In contrast, the present findings confirm the previously encountered high phenotypic similarity between Spanish and Irish *R. ponticum* (Erfmeier and Bruelheide 2005, 2010) as well as the Iberian provenance of invasive occurrences (Milne and Abbott 2000; Erfmeier and Bruelheide 2011) and, thus, suggest that mechanisms alternative to hybridization have to be considered.

### Introgression and invasion

At the molecular level, we have to completely reject the hypothesis of introgression, since diagnostic marker analysis and cluster analysis displayed no evidence of gene transfer across species. Our present analyses suggest that hypotheses based on hybridization in invasive *R. ponticum* (Cox 1979; Milne and Abbott 2000) do not apply for Ireland. In particular, Milne and Abbott (2000) found very convincing support for this hypothesis based on cpDNA and rDNA analyses conducted on 260 accessions throughout the British Isles. In their analysis, 27 of their accessions had been introgressed with genetic material from *R. catawbiense* and two further accessions displayed introgression with *R. maximum* material. In their study, the sampled *R. catawbiense* material was much more frequently encountered within Scotland than in the rest of the British Isles. However, Milne and Abbott (2000) found no evidence of introgression in the 29 accessions sampled at two locations in Ireland, which is, thus, in agreement with our data for that particular region in Ireland. Milne and Abbott (2000) suggested that the higher amount of introgression encountered in eastern Scotland might reflect a higher required cold hardiness than in other regions of the British Isles. They concluded that the increased amount of introgression might be the result of either directional horticultural selection of hardy hybrids or of natural selection after naturalization favoring individuals with higher levels of introgression from *R. catawbiense* in cold regions. Increased frost hardiness has repeatedly been invoked as an example of introgression preceding invasiveness (Cox 1979; Ellstrand and Schierenbeck 2000). In their survey on cold hardiness in the genus *Rhododendron*, Sakai et al. (1986) described that the majority of the hardiest rhododendrons belongs to the Ponticum series. On the basis of visual assessment of leaf damage after experimental frost treatment and in accordance with our results, Sakai et al. (1986) determined lowest survival temperatures of –60°C for both *R. catawbiense* and *R. maximum*, thus, ranking about the hardiest species in the genus, whereas *R. ponticum* leaves survived down to –35°C, still showing considerable frost hardiness. However, in our study, data on frost hardiness, in full accordance with the genetic data, do not support the idea of frost gene introgression into Irish populations. Given the mild temperatures and the lack of frequent winter frost events in southern Ireland (see Erfmeier and Bruelheide 2010), the need of an increased frost resistance in that part of the range does not seem to have an adaptive value. Without regularly haunting frost events the effort of maintaining frost resistance does not make sense from an evolutionary point of view (Agrawal et al. 2004). In contrast, increased frost hardiness of invasive *R. ponticum* in the northern part of the British range as assumed by Milne and Abbott (2000) resulting from directional selection and, possibly, also including introgression, is still a probable scenario that calls for a concerted testing of both introgression by means of nuclear markers and frost hardiness by means of experimental determination on a large-scale sample of populations across the British Isles.

### Trait similarities and divergences across taxa

Despite a lack of evidence of hybridization with *R. catawbiense* and *R. maximum* at the molecular level, we revealed higher similarities in some of the phenotypic responses between the invasive Irish *R. ponticum* and these North American species. In many cases, molecular markers do not necessarily reflect morphological traits or allozyme data (Hardig et al. 2000; Triest et al. 2000; Allendorf et al. 2001; Triest 2001). De Cock et al. (2003), for example, found phenotypical distinction of the hybrid taxon *Salix rubens* var*. basfordiana* from its parental taxon *S. alba* in morphological traits, although AFLP fingerprints failed to distinguish these groups. In particular, maximum germination as well as germination velocity of invasive Irish *R. ponticum* differed significantly from both conspecifics but resembled very much North American *R. catawbiense*. The most probable explanation for the similarities in germination features encountered in the present study is that similar but independent processes or chance effects have caused the observed genetic shifts. For invasive *R. ponticum*, a genetic shift in germination has been suggested before as adaptation to a more reliable environment in Ireland, lacking extreme drought or frost events during establishment in springtime, if compared to native sites in Georgia and Spain (Erfmeier and Bruelheide 2005). The relevance of differences in germination timing as trait of invasion was also demonstrated for other invasive plant species both in comparison of native and invasive species (Perglovà et al. 2009) as well as in comparisons of native and invasive populations (Kudoh et al. 2007; Hierro et al. 2009; Beckmann et al. 2011). These differences in germination traits have been shown to be heritable and to affect fitness (Leger et al. 2009), and also to be adaptive. For *R. ponticum*, a more precautious germination is of evolutionary advantage in cold regions of the native Caucasus range or under dry conditions on the Iberian Peninsula and might have been lost because of a relaxed selection pressure. Such a process can be enhanced by populations of hitherto separated origins that meet and mix (Lavergne and Molofsky 2007; Dlugosch and Parker 2008). In Irish *R. ponticum* populations, a high genetic diversity was shown to be maintained (Erfmeier and Bruelheide 2011), thus, these populations did not suffer from bottleneck effects but represent melting pot situations of increased genetic exchange. Verhoeven et al. (2011) pointed out that the benefit of admixtures is higher in the new range than at home sites because of a change in selection regime. In their review, the authors emphasize the importance of the shifted balance in costs and benefits of admixtures, and they consult a heterosis benefit as fitness boost in newly admixed populations contributing to an increased colonization success. Irrespective of inter-specific hybridization, such a heterosis effect based on admixed populations alone can account for the observed superior fitness of invasive populations (Keller and Taylor 2010; Verhoeven et al. 2011).

Relaxed selection regimes, probably supported by among population heterosis, would also explain the allocation and increased growth patterns found for Irish *R. ponticum*. The higher proportional investment in aboveground biomass is a beneficial strategy in a new, benign environment and allows for a more efficient occupancy of space, conferring a superior fitness in the face of novel habitats (Arnold and Hodges 1995). However, besides favorable abiotic conditions, the underlying cause of evolution of increased growth can also be an adaptation to open, noncompetitive environments. Blumenthal and Hubauer (2007) argue that such noncompetitive environments tend to select for traits such as rapid growth and high reproductive allocation in high resource environments. A release from competitive stress might also apply to Irish *R. ponticum* seedlings, indicated by an increased relative investment in shoot compared to root biomass. However, deciding about the relative importance of these explanations would require experimental testing of native and invasive *R. ponticum* origins in settings with different intensity levels of nutrient availability and competition.

### Conclusions on invasiveness

In the present study, we found no evidence that introgressive hybridization was involved in the evolution of invasiveness in Irish populations. In contrast, the study confirms the invasiveness due to increased germination and effective aboveground growth of Irish *R. ponticum*, suggesting that the observed phenotypic differentiation between taxa must be attributed to other driving factors. Both molecular analyses and trait analyses of seedlings revealed a 100% congruence between Irish and Spanish *R. ponticum* populations. Given the common genetic basis of Irish *R. ponticum* with its Spanish ancestors and the only moderate differentiation between Irish and Spanish seedlings in growth characteristics, the Iberian genotypes might have a similar potential of becoming invasive, too, once appropriate environmental conditions are being provided.

## Supporting Information

**Appendix S1:** Overview on diagnostic AFLP markers.

**Appendix S2:** Taxon effects of *Rhododendron ponticum* seedlings at three temperature levels.

**Appendix S3:** Overall temperature contrasts for frost hardiness (leakage rate *k*) of *Rhododendron* taxa.
