# Supplementary material for: Genetic and phenotypic differentiation between invasive and native Rhododendron (Ericaceae) taxa and the role of hybridization
Source: Ecol Evol. 2011 Nov;1(3):392–407. doi: 10.1002/ece3.38 (PMC3287310; doi:10.1002/ece3.38)
Supplement: Supplementary file 1 [file ece30001-0392-SD1.doc]

| *R. catawbiense* | *R. maximum* | *R. ponticum* | *R. ponticum* | *R. ponticum* | Number of alleles | Percentage of total number of alleles |
| --- | --- | --- | --- | --- | --- | --- |
| (USA) | (USA) | (Georgia) | (Spain) | (Ireland) |  | [%] |
| + | - | - | - | - | 10 | 2.1 |
| - | + | - | - | - | 6 | 1.3 |
| - | - | + | - | - | 6 | 1.0 |
| - | - | - | + | - | 1 | 0.2 |
| - | - | - | - | + | 1 | 0.2 |
| + | - | - | - | 0 | 11 | 2.3 |
| + | - | - | - | + | 1 | 0.2 |
| - | + | - | - | 0 | 9 | 1.9 |
| - | + | - | - | + | 3 | 0.6 |
| - | - | + | - | 0 | 10 | 2.1 |
| - | - | + | - | + | 4 | 0.8 |
| - | - | - | + | 0 | 5 | 1.0 |
| - | - | - | + | + | 4 | 0.8 |
| + | - | 0 | - | - | 15 | 3.1 |
| + | - | + | - | - | 5 | 1.0 |
| + | - | - | 0 | - | 13 | 2.7 |
| + | - | - | + | - | 3 | 0.6 |
| - | + | 0 | - | - | 11 | 2.3 |
| - | + | + | - | - | 5 | 1.0 |
| - | + | - | 0 | - | 7 | 1.5 |
| - | + | - | + | - | 1 | 0.2 |
| - | - | + | + | 0 | 9 | 1.9 |
| - | - | + | + | + | 7 | 1.5 |
| + | + | 0 | 0 | 0 | 393 | 82.2 |
| + | 0 | + | 0 | 0 | 314 | 65.7 |
| + | 0 | 0 | + | 0 | 303 | 63.4 |
| + | 0 | 0 | 0 | + | 306 | 64.0 |
| 0 | + | + | 0 | 0 | 320 | 66.9 |
| 0 | + | 0 | + | 0 | 304 | 63.6 |
| 0 | + | 0 | 0 | + | 311 | 65.1 |
| 0 | 0 | + | + | 0 | 276 | 57.7 |
| 0 | 0 | + | 0 | + | 287 | 60.0 |
| 0 | 0 | 0 | + | + | 298 | 62.3 |

Appendix S1. Overview on diagnostic markers. Numbers of private and common markers on the basis of presence/absence in the taxon and the relative proportion on the total number of alleles analysed (N=478)**.** Number of alleles was counted on different conditions of presence/absence indicated in order to identify taxon diagnostic markers, pair-wise diagnostic markers, Ponticum cluster diagnostic markers and common markers. Symbols by taxon depict: ‘+’ = obligate presence of alleles in the respective taxon in order to be counted, ‘–‘ = obligate absence of alleles in the respective taxon in order to be counted. ‘0’ = presence/ absence unconsidered in the respective taxon.
