# Supplementary figures and images for: Genetic and phenotypic differentiation between invasive and native Rhododendron (Ericaceae) taxa and the role of hybridization
Source: Ecol Evol. 2011 Nov;1(3):392–407. doi: 10.1002/ece3.38 (PMC3287310; doi:10.1002/ece3.38)

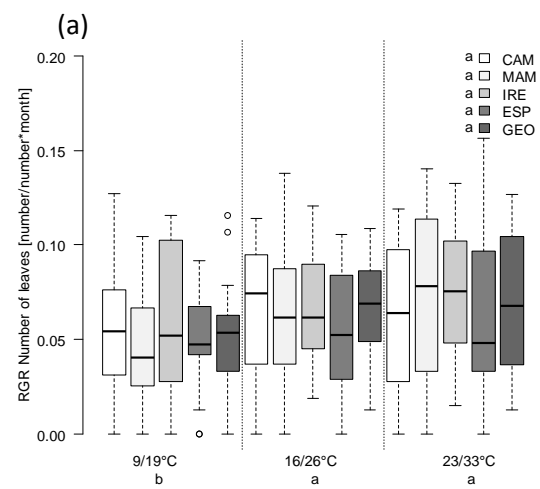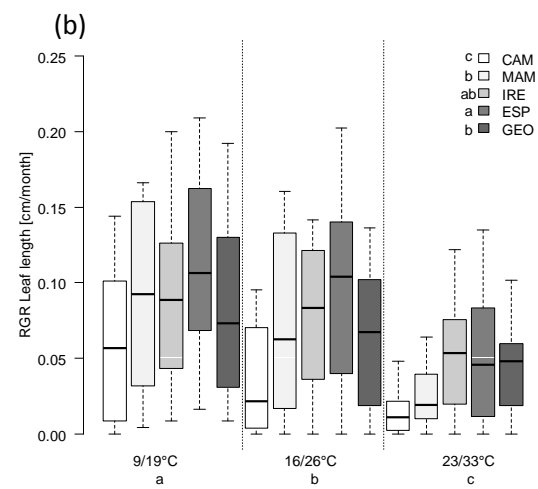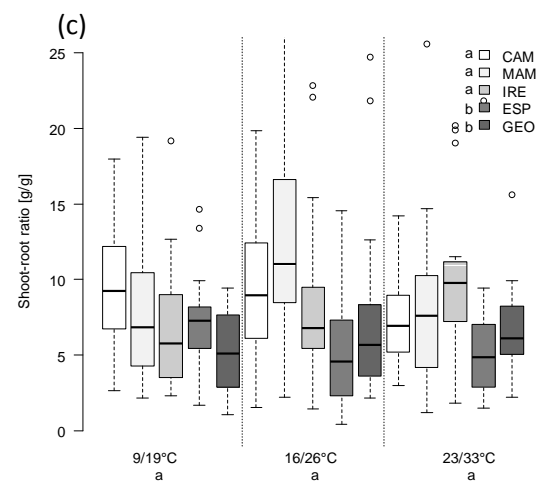

Supplement: Supplementary file 2 [file ece30001-0392-SD2.pdf]
