# Supplementary material for: Genetic and phenotypic differentiation between invasive and native Rhododendron (Ericaceae) taxa and the role of hybridization
Source: Ecol Evol. 2011 Nov;1(3):392–407. doi: 10.1002/ece3.38 (PMC3287310; doi:10.1002/ece3.38)
Supplement: Supplementary file 3 [file ece30001-0392-SD3.doc]

Appendix S3. Overall temperature contrasts for frost hardiness (leakage rate *k*) of *Rhododendron* taxa. Listed are all temperature contrasts against 4°C control.
